# Nanostructured Materials for Drug Delivery and Tissue Engineering Applications

**DOI:** 10.1007/s12033-023-00784-1

**Published:** 2023-06-22

**Authors:** Antonela Matić, Emina Karahmet Sher, Esma Karahmet Farhat, Farooq Sher

**Affiliations:** 1 Faculty of Pharmacy, University of Modern Sciences - CKM, Mostar, 88000 Bosnia and Herzegovina; 2 Department of Biosciences, School of Science and Technology, Nottingham Trent University, Nottingham, NG11 8NS UK; 3 Department of Food and Nutrition Research, Faculty of Food and Technology, Josip Juraj Strossmayer University of Osijek, Osijek, 31000 Croatia; 4 International Society of Engineering Science and Technology, Nottingham, UK; 5 Department of Engineering, School of Science and Technology, Nottingham Trent University, Nottingham, NG11 8NS UK

**Keywords:** Nanomedicine, Nanostructured materials, Tissue engineering, Controlled drug delivery, Nanoparticles

## Abstract

Nanotechnology and nanostructured materials for drug delivery and tissue engineering applications are relatively new field that is constantly advancing and expanding. The materials used are at the nanoscale level. Recently, great discoveries and applications have been made (Agents for use in chemotherapy, biological agents and immunotherapy agents) in the treatment of diseases in various areas. Tissue engineering is based on the regeneration and repair of damaged organs and tissues by developing biological substitutes that restore, maintain or improve the function of tissues and organs. Cells isolated from patients are used to seed 3D nanoparticles that can be synthetic or natural biomaterials. For the development of new tissue in tissue engineering, it is necessary to meet the conditions for connecting cells. This paper will present the ways of connecting cells and creating new tissues. Some recent discoveries and advances in the field of nanomedicine and the application of nanotechnology in drug delivery will be presented. Furthermore, the improvement of the effectiveness of new and old drugs based on the application of nanotechnology will be shown.

## Introduction

Nanomedicine is an emerging field that uses the knowledge and techniques of nanoscience in the field of medicine, remediation and disease prevention. This principle of use is manifested by the use of materials including nanorobots based on nanometric materials and nanosensors for diagnosis and drug delivery. Research has established that nanoparticles move freely and quickly in the human body compared to other materials such as drugs with very low solubility, precisely because they can be compared to particles at the atomic level. Comparing nanoparticles and other materials, it is concluded that they show better chemical, mechanical, electrical and biological characteristics than others [1]. One of the latest discoveries in the application of nanotechnology is a nanoparticle-based method that combines imaging modalities and treatments in cancer diagnosis [2–4].

The first system used in this area included lipid systems such as liposomes and micelles that were approved by the Food and Drug Administration (FDA) [5]. Due to their structure, nanostructured particles stay longer in the blood vessel system and enable the release of certain concomitant medications according to the specified dose. This procedure itself has fewer harmful effects and less damage to the plasma [3]. A relatively new and important discovery is the combination of nanoscience and bioactive natural compounds. This combination represents a great advantage in the delivery of drugs for the treatment of cancer [6]. Natural compounds are studied for their rich characteristic activities such as inducing tumour suppressor autophagy and acting as an antimicrobial agent. Their properties such as bioavailability, targeted and controlled release are greatly improved by the incorporation of nanoparticles. As an example, thymoquinone as a bioactive compound in Nigella sativa can be mentioned. Encapsulation shows a six-fold increase in bioavailability [7].

Additionally, encapsulation increases the pharmacokinetic properties, which results in better therapeutic effects. The effectiveness of nanostructures as drug carriers vary depending on their size, shape and other biophysical-chemical characteristics. Furthermore, it is necessary to carry out a lot of research on the toxicity of nanostructures to enable the safer use of these drugs. Therefore, careful design of this type of nanoparticles could be very good for further development and use. Medicines that have the characteristic of low solubility have various questions and discussions of biopharmaceutical application, including limited bioavailability after oral administration, lower diffusion capacity in the outer membrane, requiring larger amounts of intake via veins and unwanted consequences after consumption. However, all these limitations can be overcome by applying nanotechnological approaches in the drug delivery mechanism [8].

This paper will present some research that has been carried out so far regarding the application of nanoparticles in drug delivery and applications in tissue engineering. In particular, referring to the works published by Kumar et al. [9], Sharma et al. [10] and Portero et al. [11] in which successful and unsuccessful attempts to form nanocarriers with certain particles in the process of controlled drug release are described. Looking back at all the works published so far on this topic, it is noticed that so far natural medicines have not been sufficiently researched as carriers in the delivery of medicines. The fact is that recently more and more research is being imposed based on controlled drug delivery, so it is necessary to make an overview with summary research on this issue. This paper presents summarizing the database of research carried out so far to enable easier further research. Especially looking at drug delivery in tissue engineering will be presented in this paper.

## Nanotechnology

Today, nanotechnology is a multidisciplinary field based on the study of processes that deal with nanometer-sized particles, as well as on the design, synthesis and use of nanometer-sized materials. It is the biggest challenge of the twenty-first century. Today, nanoparticles and nanopowders are known in the form of aerogels, sol-gels and colloids. The very diversity in their production enables a rich selection of production of new materials. Nanomaterials are materials with dimensions less than 1000 nm in length and 100 nm in diameter. There is no unique standardization of nanomaterial size for the USA and Europe. They differ in their shape and physical and chemical properties and there are many divisions of nanomaterials. Nanoparticles and nanomaterials are often equated. The difference is that nanoparticles are just one type of nanomaterial that has a spherical shape and three dimensions [12, 13].

### Nanostructured Materials

The standardization and division of already known nanomaterials have not yet been officially approved, so the exact distribution by category is not yet known. According to world scientists, it is believed that in the next ten years, a decision will be made according to which standardization will be distributed. Although there is no single and unequivocal distribution, nanomaterials have been divided into groups throughout history. The main distribution refers to the structure and shape of the nanostructured materials themselves. They are divided into two groups, namely dispersions and solid (Joined) nanomaterials. If dispersed systems are known, the division of nanomaterials is also known. Solid nanoparticles are tiny, lipid, spherical formations 40–1000 nm in size. It is solid at room temperature, easily produced without organic solvent, easily sterilized, very stable and has a high capacity for drug molecules. The solid-state allows gradual delivery of the drug. The basic division is into natural and artificial nanomaterials [14, 15].

Natural nanomaterials include materials with biological systems, e.g. viruses [7]. The term artificial nanomaterial refers to those materials that are produced by various technological processes. Nanomaterials can also be divided according to dimensionality, as given in Table 1. 0 D and 1 D nanomaterials have very similar characteristics and they are: monocrystalline and polycrystalline, shapes like spheres and balls, metal, polymer or ceramic. 2.D nanomaterials are characterized by properties: built from one or more chemical elements, metal, polymer, ceramic, single-layer or multi-layer and crystalline or amorphous. 3D nanomaterials can be divided into groups and they are: built from carbon, made of metal, composites and dendrimers [16]. Based on the bulk material from which nanomaterials and nanoparticles are made, they can be divided into four categories, as given in Table 2 [13].

**Table 1:** Division of nanoparticles according to dimensionality [16, 17]

Dimensionality	Type of nanomaterial
0D nanomaterials	Nanoparticles, quantum dots, nanocrystals and clusters of crystals
1D nanomaterials	Nanotubes, nano gels, nanofibers and polymer chains
2D nanomaterials	Nanofilms, nanolayers and nanocoatings
3D nanomaterials	Nano gels, nanotubes, nanoparticle fullerenes or nanolayers

**Table 2:** Types of nanoparticles according to material types

Bulk material	Morphology
Carbon-based	Hollow tubes, ellipsoids or spheres, fullerenes (C60), carbon nanotubes (CNTs), carbon nanofibers, carbon black, graphene (Gr) and carbon onions
Inorganic-based	Metal and metal oxides, such as Au or Ag NPs, metal oxides such as TiO_2_ and ZnO NPs and semiconductors such as silicon and ceramics
Organic-based	Dendrimers, micelles, liposomes and polymer nanomaterials
Composite-based	Hybrid nanofibers, metalorganic frameworks, combinations of carbon-based, metal-based, or organic-based nanomaterials with any form of metal, ceramic or polymer bulk materials

According to their origin, nanomaterials are divided into two subgroups, natural and synthetic. Natural nanomaterials are made by the forces of nature. They can be found in the atmosphere and on the ground. Synthetic nanomaterials are produced by different methods such as physical, chemical, biological or hybrid methods [13]. Nanomaterials have different properties and are significantly improved compared to bulk materials due to the perfect crystal lattice [18, 19]. They have better characteristics compared to the raw material from which they are made, better durability and flexibility. For example, nanomaterials with metals such as copper and silver have excellent optical properties. Nanomaterials with copper have a high absorption potential. Ferrofluid has strong magnetic properties [13]. Gold nanoparticles are red or purple, compared to gold which is normally yellow. The properties of nanomaterials and nanoparticles can be tuned by changing the size and morphology. Absorption and emission of light directly depend on it [18, 19]. Important characteristics of nanomaterials and nanoparticles are a large surface area compared to the raw material, the occurrence of a magnetic field, high mechanical resistance and antimicrobial properties (Antibacterial, antiviral and antifungal) [18].

### Synthesis Process of Nanomaterials and Nanoparticles

There are two approaches for the synthesis of nanomaterials marked as bottom-up and top-down approaches. A top-down approach represents a group of different processes in which larger molecules are degraded into smaller ones and then used as a base for nanoparticle formation, i.e. the breaking of the microstructure in the already existing nanostructure. This approach includes the following procedures: mechanical grinding, strong plastic deformation of the sample and lithography. It also involves processing the bulk material, crushing and obtaining finer particles using mechanical grinding, laser ablation, etching, sputtering and electro-explosion. Mechanical grinding is an effective and most often used bottom-up method for obtaining nanocomposites, aluminium alloys with oxide and carbide, nanoalloys of aluminium, nickel, magnesium and copper. It enables the transfer of energy, obtained from the high-energy steel ball or tumbler to the powder.

This powder then creates shear stress and enables the formation of nanomaterials. Another used procedure in the bottom-down process is thermal decomposition, an endothermic procedure in which the decomposition of larger molecules is enabled by the use of heat. The nanoparticles are produced during this process at a specific temperature. Lithographic methods are also another example of bottom-down methods. Nanoimprint lithography uses produced template materials on which the soft polymeric material is stamped to create a specific pattern. By the use of latex sphere, a templated matrix is produced by different lithography techniques, such as electron beam lithography, nano-imprint lithography and photo-lithograph [20, 21].

The bottom-up approach is characterized by joining atom by atom or layer by layer to obtain a nanomaterial. The bottom-up approach includes several methods of aggregating particles from the level of atoms and molecules, through nuclei, to nanoparticles. There is a wide palette of methods used to aggregate particles to the size of nanoparticles and nanomaterials, such as gas condensation, mechanical grinding, supercritical fluid synthesis, spinning, sol–gel process, laser pyrolysis, chemical vapour deposition, molecular condensation, chemical reduction and green synthesis [18]. In theory, the ideal procedure would be to directly move atoms and molecules, but this is not simple in a real system because it is currently impossible to apply such a force on such a small material [22].

The oldest bottom-up method used in the production of nanoparticles is gas condensation. The method involves heating metal or inorganic materials in a refractory vessel, under a pressure of 1–50 mbar. During the evaporation of the gas, pressure is created, whereby the metal ions collide with the gas and metal nanoparticles accumulate. Metal nanoparticles are removed with a metal scraper. It is important to match gas and metal compatibility. In addition to thermal, laser vaporization can be used. The most often used process for the synthesis of nanoparticles, due to its efficiency and simplicity, is marked pyrolysis. The starting precursor can be liquid or vapour and it is transformed into nanoparticles by the process of burning in flame or heating by the use of laser and plasma at high pressure. Another method of synthesis of nanoparticles is spinning. In this process, the spinning disc reactor (SDR) is used, which is a rotating disc with controlled physical parameters. The electric force is applied and charged threads of polymer solutions melt, producing nanofibers with specific properties [23].

### Nanomedicine

Nanomedicine is a relatively new concept in the field of science and medicine itself. It is a branch of medicine based on the medical application of nanotechnology through the application of nanoparticles, nanoelectronic biosensors and molecular nanotechnology. A special part of nanomedicine refers to nanomedicine aimed at tissue engineering. This branch will enable the complete replacement of damaged tissues with new tissues. It is believed that in the near future nanomedicine will enable the restoration of old and the production of new tissues and that in the future it will not be necessary to look for donors to donate certain tissues, but it will be possible in this direct way. The size order of nanoparticles is very similar to the size order of most biological molecules and structures, therefore nanoparticles can be very useful in both in vivo and in vitro biomedical research and applications. Nanomedicine has brought the greatest progress and merit in the delivery of drugs to a specific cell with the help of nanoparticles [24].

Communication between medicine and nanotechnology enables a better understanding and treatment of the living system, the synthesis of drugs and their targeted delivery to the diseased cell. Nanomedicine is based on three main parts: (1) Nanostructured materials and devices that promise success in advanced diagnostic biosensors, targeted drug delivery and smart drugs; (2) benefits of molecular medicine through genetics, proteomics (Proteomics deals with the study of proteins that carry all biological functions and are targets for drugs) and artificially produced microorganisms; (3) Molecular-mechanical systems such as nanorobots that would enable immediate diagnosis with the destruction of the cause of pathology, chromosome replacement, individual cell surgery in vivo and effective progress in natural physiological functioning [25].

### Nanotechnology in Drug Delivery Process

Drug delivery can be defined as a procedure that enables the pharmacologically active substance to reach the in vivo location while at the same time minimizing toxicity. The development of particles or molecules of the order of the size of nanoparticles improves the bioavailability of drugs, improves solubility and different dosages of drugs or is used to research a better mechanism of action of the drug [26]. Bioavailability of the drug implies the presence of the drug in the place where it is needed and where it will be most useful in the body. Considering that, the delivery of drugs using nanoparticles is based on maximum bioavailability at specific places in the body and at a certain time. This process is enabled by molecular targeting using neuro devices [27]. The strength of a drug delivery system is based on the ability to alter pharmacological distribution and biodistribution. Nanoparticles enable the delivery of drugs precisely because of their order of size, because the cells accept them, while a larger particle would be considered a change and would reject them. Nanotechnology aims at changing the formulation of compounds and extending the life of already existing compounds [27].

### Nanoparticles as Drug Carriers

The advantage of nanoparticles as drug carriers is characterized by high drug concentration at the target site, control of drug release kinetics, a simple modification of the particle to monitor target cavities and surfaces, protection of the drug from enzymatic degradation and preferential release of the drug in the target tissue. Nanoparticles are used for pharmacological purposes and can be classified into certain groups and they are non-polar nanoparticles, metal nanoparticles, oligomeric micelles, dendrimers, inorganic nanoparticles and nanocrystals [9]. The following series will present the most significant examples of research that has been carried out so far based on the distribution of certain groups of nanoparticles that are possible for use in targeted and controlled drug delivery.

#### Bipolar Nanoparticles

The most significant example of bipolar nanoparticles that have been investigated so far is chitosan. It is also known as chitosan in the pharmaceutical industry. This nanoparticle shows properties that can be characterized as an adhesive on the target molecule and can be used to act in tight parts of the human body. Chitosan-based nanoparticles are used for systemic drug release for different types of epithelial tissue and some examples are buccal epithelium [28], nasal epithelium [29], eye epithelium [30] and lung epithelium [31]. Silva et al. [32] evaluated the efficacy of a 0.75% isotonic solution of hydroxypropyl methylcellulose (HPMC) containing chitosan/sodium tripolyphosphate/hyaluronic acid nanoparticles for delivery of the antibiotic ceftazidime to the eye. The nanoparticles showed adhesion that gave good results with the mucosa of the eye and gave a long time for the release of the antibiotic.

From this, it can be concluded that nanoparticles are able to improve the lifetime of the drug in the eyes. Pistone et al. [33] prepared nanoparticles of chitosan, alginate and pectin as potential candidates for drug delivery in the oral cavity. The biocompatibility of this formulation was evaluated based on the solubility of chitosan, which was very unstable during 2 h in artificial saliva. Alginate particles proved to be the most pronounced in this experiment. However, considering the concentration of alginate and pectin particles, they showed cytotoxicity and chitosan particles were the most competitive to cytologists. From all this, they concluded that each particle showed some advantages, but also some limitations for the use of drug delivery and further research is needed. In one application reported by Jain and Jain [34], 5-fluorouracil (5-FU) from hyaluronic acid coated with chitosan nanoparticles was investigated. Oral administration in conditions simulating transit from the stomach to the large intestine released 5-FU. A high local concentration of drugs could increase the delivery time and thereby improve the effect on tumour cells.

#### Metal Nanoparticles

A special interest from the medical side has arisen in the research of metal particles. The demand for such particles is clearly arising due to processes such as biosensors, targeted drug delivery, hyperthermia and certain therapies [35]. The functionality of metal nanoparticles enabled the binding of ligands, drugs and antibodies. Based on this functionality, these particles provide a promising application in biomedicine [36]. Most research on metal nanoparticles has been conducted on silver, gold, copper and iron. Recently, efforts have been made to investigate other metal nanoparticles as well [36]. Other metals that are possible to use in nanoparticles are cadmium, platinum, zinc, selenium and sulfur. To incorporate these metal ions into nanoparticles, appropriate conditions of temperature and pressure are needed as well as enzymes from eukaryotic and prokaryotic cells [37, 38]. The metal used for nanoparticle production mustn't be harmful and toxic to animals and humans nor given any type of adverse reaction [39].

#### Polymer Micelles

Polymeric micelles are built from a copolymer consisting of a hydrophobic and a hydrophilic monomer unit. The hydrophobic core can be used to store hydrophobic drugs and the hydrophilic core can be used to store hydrophilic drugs. The release of the active substance from the micelle is influenced by: the diffusion rate of the active substance, the distribution coefficient, the physicochemical properties of the active substance, the position of the substance inside the micelle and the rate of copolymer decomposition. The release of substances from the micelle can also be influenced by external stimuli such as pH, light, temperature, ultrasound, magnetic fields and light (UV, infrared or visible) [40]. Research has shown that polymer micelles increase permeability and thereby increase the concentration of the active substance in tumour tissue. The mechanism for this type of action is known as the mechanism of passive targeting [40].

**Fig. 1:**
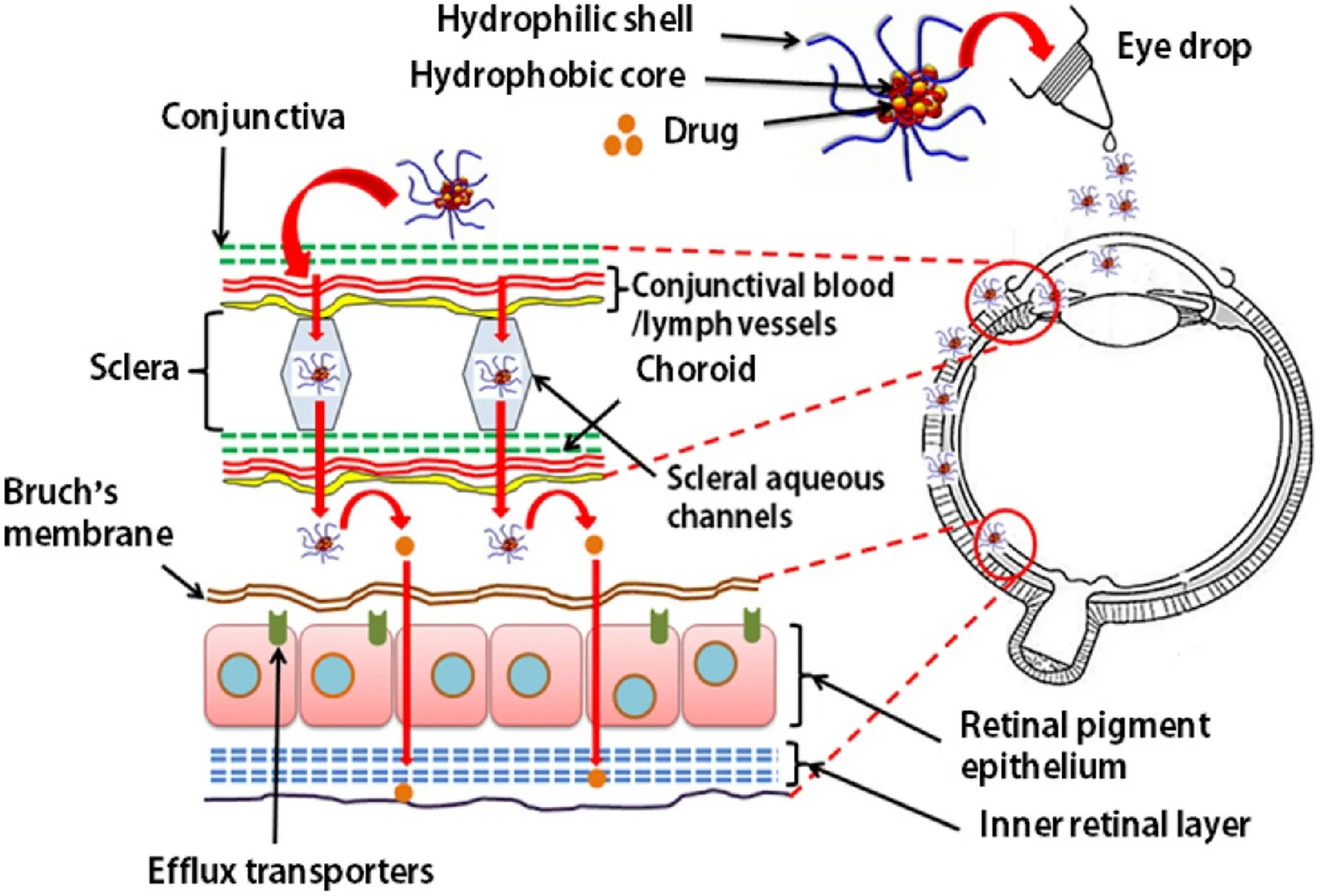
Possible positions of the drug in the micelle; drug delivery to the eye [9]

The drug is delivered inside the micelle in three known ways, namely: the dissolution process by direct action, the evaporation-based process and the dialysis process. The direct dissolution procedure takes place in such a way that the copolymer and the drug are combined in an aqueous medium and form a drug filled with micelles. In the solvent evaporation process, the copolymer and the drug are dissolved using a volatile organic solvent. The dialysis process is a little longer because separate dissolution procedures take place. The drug is dissolved in one solvent and the copolymer in another solvent. After dissolution, the process of combining the dialysis bags occurs and then the dialysis process creates micelles [41]. Figure 1 shows the possible position that the drug can occupy in the micelle. The direct dissolution procedure takes place in such a way that the copolymer and the drug are combined in an aqueous medium and form a drug filled with micelles.

**Fig. 2:**
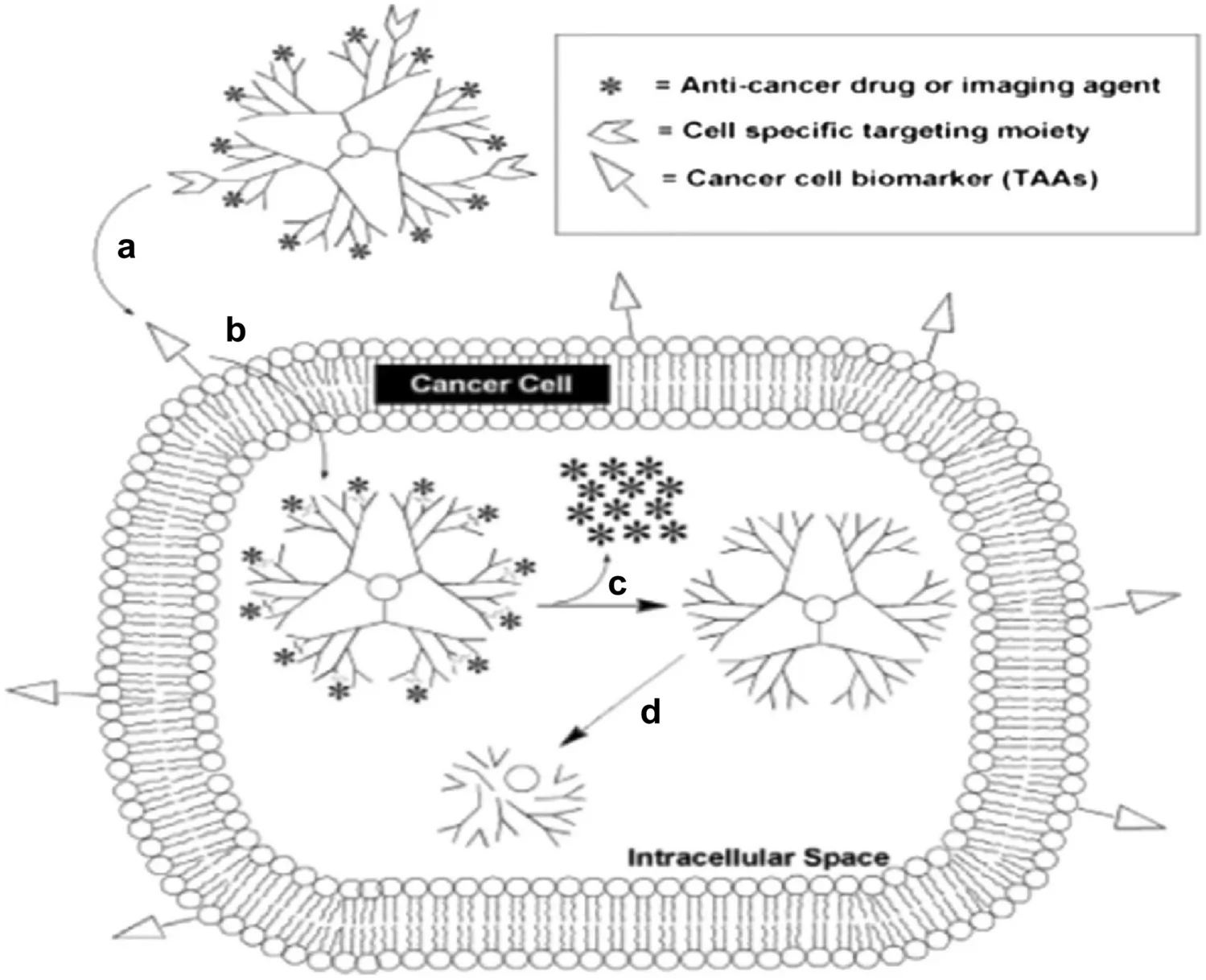
Drug delivery using a dendrimer; **a** Representation of dendrimers with surface multiple functions that can be targeted to cancer cells that have anti-tumour antibodies in their composition, **b** Entry into the cell by endocytosis, **c** Upon entering the cell and **d** The drug is released from the dendrimer disintegrating the dendritic scaffold [45]

Dendrimers are perfectly ordered, monodisperse, three-dimensional polymers with a characteristic structure originating from a central core. They are less than 100 nm in size and have the ability to bind ligands to their branches. Dendrimers can be synthesized by two approaches: Creation from the core outwards and Convergent–which starts from the outside [42]. Given that they retain amine groups in their structure, dendrimers are limited in clinical application. The groups are positively charged, which makes them toxic. Encapsulation of the drug in dendrimers can take place through three mechanisms, namely: simple encapsulation, electrostatic encapsulation and covalent encapsulation [43]. So far, two ways of releasing the drug from the dendrimer have been proven: In vivo by breaking down the covalent bond based on available enzymes or by cleaving the bond based on a favourable environment and drug release due to changes in the physical characteristics of the environment, such as pH-value, temperature, etc. [43]. Jain et al. [44] described poly-L-lysine as a very good drug delivery principle for cancer prevention. Figure 2 shows an approach to the request for the delivery of dendrimer-based cancer drugs.

#### Inorganic Nanoparticles

Research on inorganic nanoparticles is not as frequent as other areas of nanoparticles, but again there are a few recorded examples. The inorganic nanoparticles investigated so far are silver, gold, silicon and iron oxide. Metal particles such as gold and silver have a special property–surface plasmid resonance (SPR). Research on their activity in the field of drug delivery could not explain whether the ionized form is related to their toxicity. As for their in vivo transport, not a single confidential study or record was found [46]. In one of the papers, information was found that drugs can be bound to the surfaces of gold nanoparticles through ionic or covalent bonds or physical absorption. Their release is possible through biological stimuli or light activation [47]. Silver has very little recorded research on drug delivery. Prusty and Swain [48] synthesized a polyacrylamide/dextran hybrid system of nanohydrogels with covalently bound silver nanoparticles for the release of ornidazole. It has been proven to have an in vitro release of 98.5% [48].

#### Nanocrystals

Nanocrystals are nanosized particles of poorly soluble active substances. They are less than 1000 nm. This procedure for the preparation of nanoparticles is done because, due to the reduction in the size of the solid particle of the active substance, the number of particles increases and thus the specific surface area of the solid increases. By increasing the surface area of the solid, the diameter of the solid particle decreases. The rate of dissolution of nanosized substances is much higher than the rate of dissolution of micronized solids precisely because of the increase in the surface area of the solid. Namely, it is already known that the larger the solid surface is, the better the dissolution rate [49]. Ni et al. [50] showed the incorporation of substances based on nanocrystalline structure into chitosan for the possibility of delivering hydrophobic drugs.

## Nanosystems for Drug Delivery

**Fig. 3:**
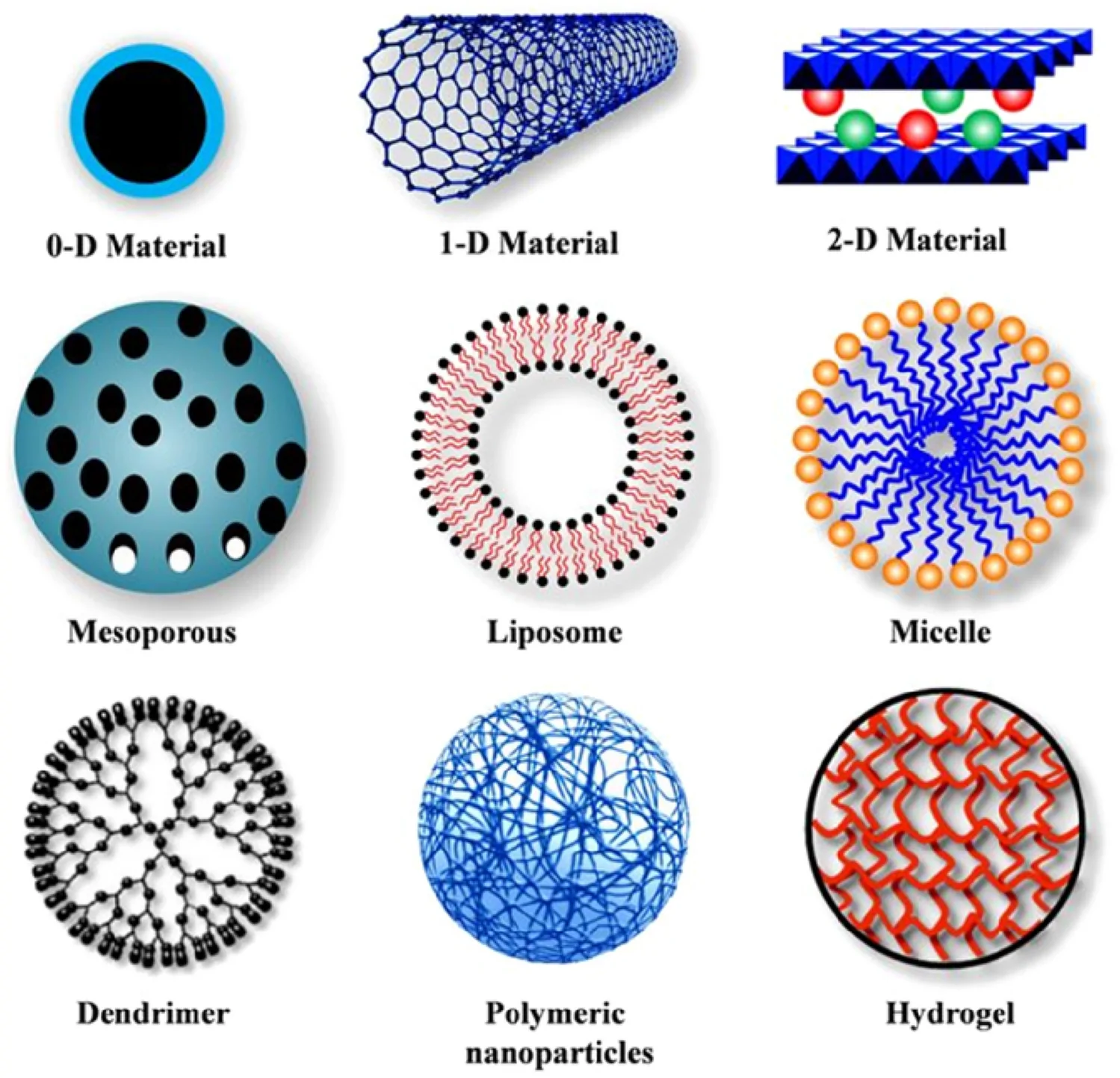
Schematic representation of certain main types of nanoparticle formulations used in the drug delivery system [52]

A nanosystem for drug delivery is built from a core or emulsion of particles to serve as a carrier. The particles in the core are prepared by chemical methods to enable this procedure. Functional groups are added to the core, which can be therapeutic molecules or ligands for targeting specific locations. For the delivery system to be successful, it must be characterized by the following features: optimal dosing properties, optimal drug release properties, long time of systemic circulation and low toxicity [51]. Figure 3 shows a schematic representation of several main nanoparticle formulations used in the drug delivery system.

### Drug Delivery Design and Process

The process of the advancement of nanotechnology in the drug delivery system is based on the research of new ways of drug administration and special emphasis is placed on this, ensuring their targeted action in certain regions, thereby reducing toxicity and increasing bioavailability in the body [53]. Advances in sciences based on computer processes and experiments for the categorization procedure, the purification procedure of proteins and peptides of biological targets are the most significant for the improvement of this sector [54]. As an example, Chen et al. [55] explained and demonstrated the use of nanocarriers for imaging and sensing applications. Pelaz et al. [56] presented a very good example of the application of nanoparticles in nanomedicine and discussed new research and challenges in this area. Mattos et al. [57] showed that the release profile of silica nanoparticles grafted with neem bark extract was lower than that of biogenic silica nanoparticles with neem bark extract.

**Fig. 4:**
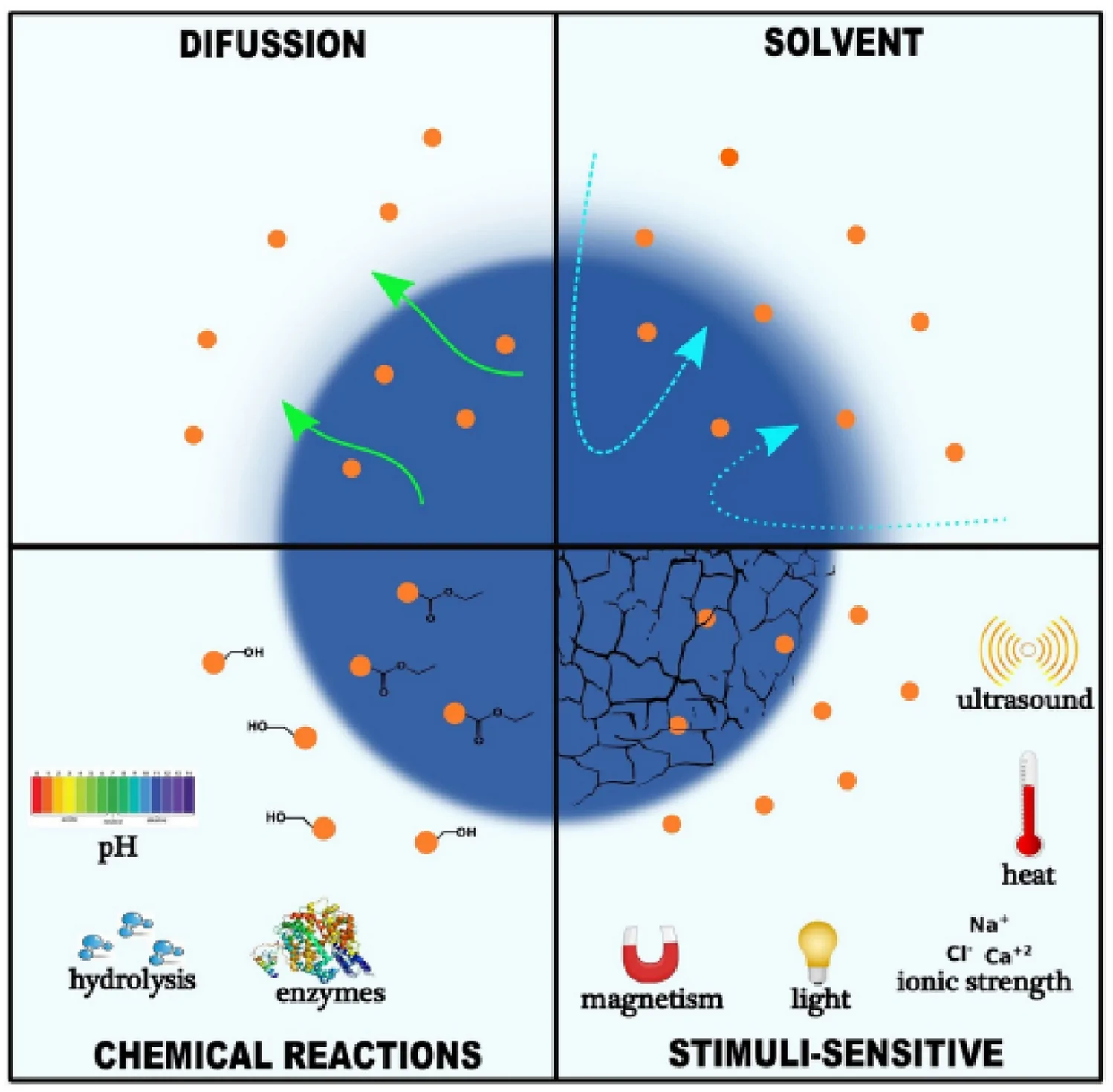
Drug release mechanisms- diffusion, solvent, stimuli-sensitive and chemical reaction [9]

All the mentioned facts affect the communication of nanocarriers with the organ system [58], but also the speed of release of active substances into the body [59]. A group of authors [60] considered the so-called multiple lipid shell (CLS) constructed of docetaxel and wortmannin as drug prototypes used to control drug release kinetics. They also studied the discharge profile which they obtained favourably and affected both in vivo and in vitro conditions. To understand the drug delivery profile, it is required to take into consideration the composition of the nanocarrier (Organic origin, inorganic origin or hybrid materials) and the form in which the drugs are connected to it (Core–shell system) [61]. The mechanism of drug release can be represented by mechanisms such as diffusion, chemical reaction, stimulus-controlled release and solvent. This method is shown in Fig. 4. [62]. Kamaly et al. [63] gave a detailed insight into controlled drug release from polymeric nanoparticles. So far, several nanoparticles with different drug release profiles have been discovered, tested and approved. Currently, efforts are being made to design a better system with specific characteristics of nano culture for targeted regions in the organism [64–66].

Most efforts are made to reduce immunogenicity utilizing coatings for certain substances, such as polymers [67], natural polysaccharides [68], antibodies [69], cell membranes [70]. Ligand-modified nanocarriers pass through the cell membrane and in this way manage to deliver the drug in a programmed manner to a precisely determined environment. As an example of this carrier, hyaluronic acid can be mentioned. In the role of a ligand attached to a nanocarrier, it showed very good results in strengthening the antitumor effect against sites similar to melanoma [71]. This research showed promising results, but when developing and forming a system for drug delivery via ligand binding to a nanocarrier, it is necessary to perform a lot of checks and pay attention to a lot of factors such as blood flow variables, the stage of the disease and the composition of the tissue itself [72].

So far, the ligands that have been used have been examined in terms of ligand-nanocarrier interaction, but the delivery mechanism ie. drug release is still unclear. Stimulus-sensitive nanocarriers are capable of controlled drug release with the help of external factors such as ultrasound [73], heat [74], pH value [75], ionic strength [76], light [24] and magnetism [77]. As an example, the super magnetic iron oxide Fe_2_O_3_ can be mentioned. Oxide particles are connected to the polymer carrier and are released into the system by the action of a magnetic field [78]. Particles for use in chemo-photothermal therapy were synthesized based on Au/Fe_3_O_4_ polymer nanoparticles [79].

## Tissue Engineering

**Fig. 5:**
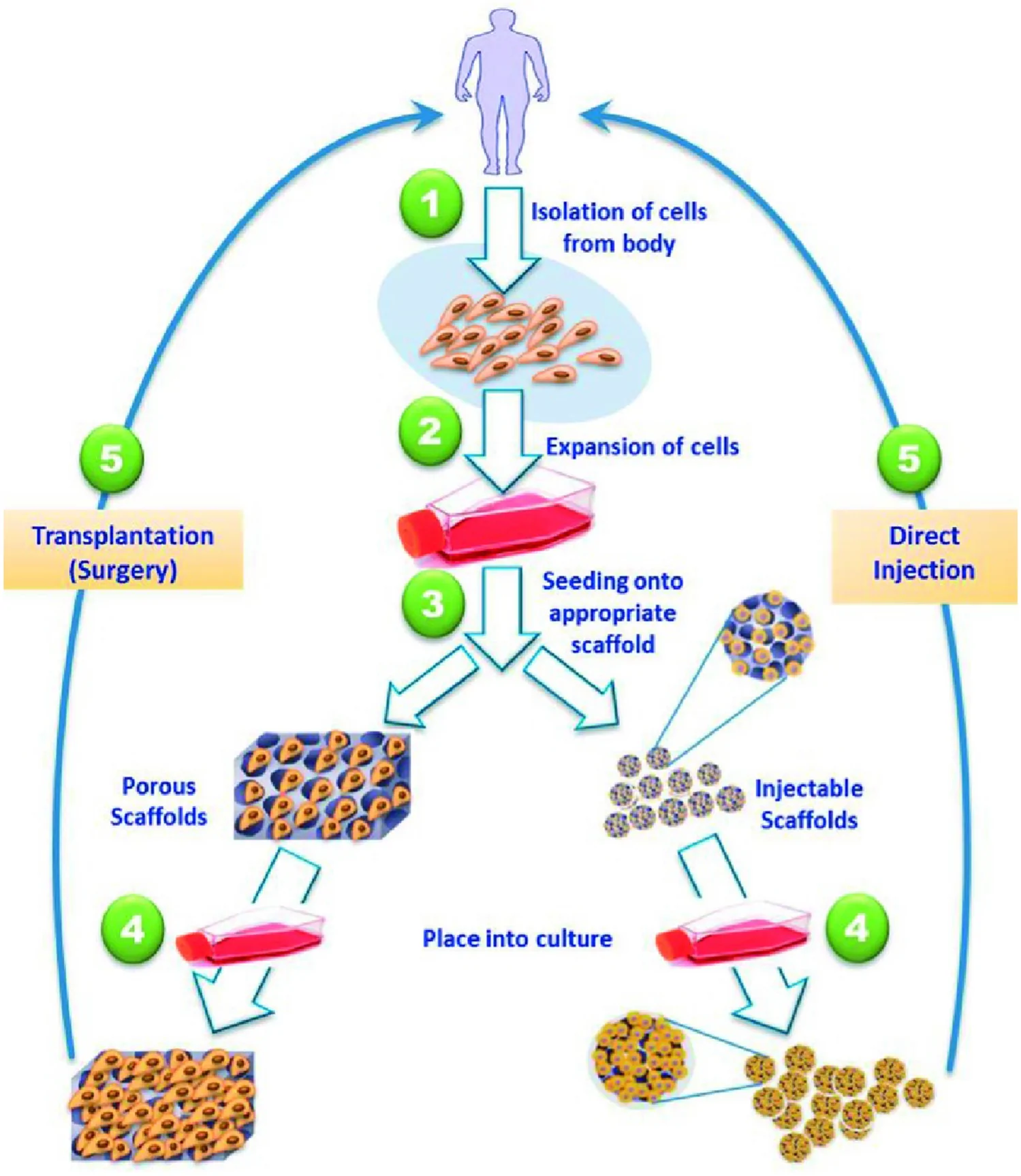
Major steps in tissue engineering; The specific cell that will be used for tissue engineering is isolated from the patient’s sample, captured in vitro, incorporated into a newly designed scaffold and transplanted into the patient by injection [80]

Tissue engineering is a relatively new field whose main goal is the development of biological substitutes that can restore, maintain or improve the function of a diseased or damaged cell or tissue. To achieve better results, tissue engineering triads are formed. A carrier is used as a template for the creation of new tissue that is implanted in cells isolated from biological tissue and exposed to various biophysical stimuli in a bioreactor. The main steps of tissue engineering are shown in Fig. 5. [80]. Carriers used in tissue engineering must meet several conditions. The first condition is biocompatibility. Cells must adhere to the material, function normally, migrate across the surface and through the support and begin to multiply (Proliferate) on the material before depositing a new extracellular matrix. Therefore, tissue engineering is based on the principle of developing novel tissues with improved features when compared to the original tissues using artificial scaffolds and cells.

However, both natural and synthetic materials have faced many obstacles when used as scaffolds during this process, such as the development of their mechanical features and unique composition, avoidance of degradation by host tissues and biocompatibility and interplay with tissue niche. Therefore, the use of nanomaterials has given some new perspectives in the field of tissue engineering and in developing novel artificial scaffolds, as well as in enabling them to connect into complex hierarchical systems that mimic the organization of cells in the tissues in the human body. For example, the electrospinning of fibrous matrices promotes cell proliferation and differentiation, allowing them to form complex networks in the extracellular matrix. In addition, this process is controlled by nano-scale topography [81]. In addition, different features of nanoparticles or nanofibers used in tissue engineering can be altered by a range of chemical and physical modifications, which allows them to connect with host cells. For example, artificial scaffolds are often used in bone tissue engineering as providers of mechanical support, as well as the creators of an optimal environment for bone regeneration. Scaffolds based on polymers are often used and should have both adequate mechanical features and robustness, as well as the surface characteristic that enables cell adhesion and differentiation. Their properties are enhanced by the use of nanomaterials as fillers, such as carbon-based and metal-based nanomaterials. Therefore, the incorporation of nanomaterials in the development of artificial scaffolds increases the success of tissue engineering processes [82].

In addition, tissue engineering is closely related to the delivery of a drug or specific molecules to a specific targeted site by the use of nanomaterials that also improves features of artificial tissue scaffolds. To deliver pharmacologically active substances, nanomaterials need to enter the target places in the body which sometimes can be challenging due to gut microbiota and its influence on drug absorption [83]. The mechanism of entry of nanomaterials into cells corresponds to endocytosis. Considering that endocytosis implies fusion with lysosomes, nanomaterials should be resistant to low pH and lysosomal activity. For small-size nanomaterials, the mechanism of entry is called pinocytosis and for particles larger than 500 nm, the mechanism is called phagocytosis. Pinocytosis can be further divided into clathrin-mediated endocytosis, caveolae-mediated endocytosis and clathrin- and caveolae-independent endocytosis. A characteristic of clathrin-mediated endocytosis is the recycling of material from which vesicles are formed to transmit signals and nutrients.

In this way, nanomaterials that are not digested by macrophages, such as nanoparticles of silver and titanium dioxide, are introduced into the cell and it plays an important role in maintaining cellular homeostasis and signalling regulation. The protein component clathrin binds to specific sites in the cytosol. During the transport of nanoparticles in the vesicle, a reaction occurs between the nanoparticle and the adapter. As a result, phosphorylation or ubiquitination of the cargo protein occurs [84]. Caveolae-mediated endocytosis is intended for nanoparticles with a diameter of 50–60 nm, such as proteins, fatty acids and lipids. Caveolae vesicles are specific in that they can merge into one larger particle and thus avoid degradation by lysosomes. This way of entering the cell is also used by viruses. Endocytosis independent of clathrin and caveolae can also be called macropinocytosis. It serves to transport macroparticles of nanomaterials that cannot be transported in any other way. Nanomaterials and nanobiovectors enter cells via endocytosis, usually mediated by receptors on the cell surface. They achieve this thanks to the positive charge they have and easily gravitate toward negatively charged cells [84].

To deliver pharmacologically active substances, nanomaterials need to enter the target places in the body. The mechanism of entry of nanomaterials into cells corresponds to endocytosis. Considering that endocytosis implies fusion with lysosomes, nanomaterials should be resistant to low pH and lysosomal activity. For small-size nanomaterials, the mechanism of entry is called pinocytosis and for particles larger than 500 nm, the mechanism is called phagocytosis. Pinocytosis can be further divided into clathrin-mediated endocytosis, caveolae-mediated endocytosis and clathrin- and caveolae-independent endocytosis. A characteristic of clathrin-mediated endocytosis is the recycling of material from which vesicles are formed to transmit signals and nutrients. In this way, nanomaterials that are not digested by macrophages, such as nanoparticles of silver and titanium dioxide, are introduced into the cell, and it plays an important role in maintaining cellular homeostasis and signalling regulation. The protein component clathrin binds to specific sites in the cytosol. During the transport of nanoparticles in the vesicle, a reaction occurs between the nanoparticle and the adapter. As a result, phosphorylation or ubiquitination of the cargo protein occurs [84]. Caveolae-mediated endocytosis is intended for nanoparticles with a diameter of 50–60 nm, such as proteins, fatty acids and lipids. Caveolae vesicles are specific in that they can merge into one larger particle and thus avoid degradation by lysosomes. This way of entering the cell is also used by viruses [84]. Endocytosis independent of clathrin and caveolae can also be called macropinocytosis. It serves to transport macroparticles of nanomaterials that cannot be transported in any other way [84]. Nanomaterials and nanobiovectors enter cells via endocytosis, usually mediated by receptors on the cell surface. They achieve this thanks to the positive charge they have and easily gravitate toward negatively charged cells [84].

After being incorporated into the body, the carrier must not cause an immune reaction, that is, an inflammatory process that could reduce healing or cause the body to reject the material. One of the goals of tissue engineering is to enable the patient's cells to replace the implanted carrier over a certain period by creating their extracellular matrix. Therefore, the carrier should be fully biodegradable and break down at a controlled rate of degradation that matches the rate of new tissue formation. By-products of biological decomposition must not be toxic and must leave the body without interactions with other organs. The support should have mechanical properties that correspond to the anatomical position of installation and ensure easy handling during the surgical procedure [52].

The structure of the carrier is extremely important. Carriers must have a highly porous structure with well-interconnected pores to ensure cell migration and ingrowth of new blood vessels (Vascularization) throughout the entire volume of the carrier and diffusion of nutrients to cells within the carrier and waste products from the carrier. Pore size is also very important. Interactions between cells and carriers are primarily achieved through chemical groups (Ligands) whose density depends on the available surface. The pores must be large enough so that cells can migrate into the support structure and bind to the ligands inside the support, but also small enough to provide a large surface area and the appropriate density of ligands needed to bind a sufficient number of cells to the support. Different types of cells and tissues require different ranges of pore sizes in tissue engineering supports [80].

Tissue engineering and nanomedicine find practical applications in the treatment of many bone diseases. Some of them are loss of bone tissue due to trauma and accidents, cancer of bone tissue, bone injuries caused by firearms, amputation of limbs and osteoporosis. The excellent integration of nanoparticles in these cases gives promising results. Ceramic nanoparticles are most often used for bone regeneration, i.e., poly (Lactic-co-glycolic) acid (PLGA), gelatin, collagen and chitosan. Bisphosphonate drugs used for the treatment of osteoporosis have a high affinity for bone tissue. They can serve to connect regenerating nanoparticles and bone minerals. The synergy of bisphosphonates and regenerating nanoparticles results in a targeted therapy to promote bone tissue regeneration. Several different agents (Those that promote osteoblast activity and those that inhibit osteoclast activity) can be added to targeted therapy of tissue engineering [85, 86].

### Course of Tissue Engineering Development

For tissue engineering to have a successful outcome, it is necessary to satisfy three main components. They are known as the tissue engineering triad” and consist of scaffolds, cells and growth factors [87]. At the very beginning of the development of this technology, the greatest care was taken in the selection of the material to be used for the scaffolding. With further development, tailoring the surface functionality led to the mimicking of the extracellular matrix using a scaffold and this greatly helped. Replacing the two-dimensional structure with nanometric materials led to better and more significant results. The next generation in tissue engineering research is based on growth factor technology because it was realized that a certain amount of chemical factors at the right time was sufficient to maintain the functionality of a seeded cell at scale.

Such attempts at tissue engineering were very successful for cartilage and bone tissue. Special attention should be paid to tissue engineering and it should be emphasized that the possibility of tissue regeneration depends greatly on the type of cell and the age of the cell itself [88]. The third generation of tissue engineering constructs used stem cells at scales with nanostructured materials including the appropriate growth factor to promote specific cells into specific lineages. This procedure serves to expand the application and the nanostructured platform for the regeneration of certain types of tissue whose cells originate from the same germ leaf. In recent years, the fourth generation of tissue engineering has emerged that seeks to improve functional tissue regeneration. Until now, it is known that the functional expression of the cell is determined by the pressure and mechanical forces acting on the cell. Such functional expression is achieved by activating cellular signalling pathways and the process itself is called mechanotransduction. Such a discovery resulted in the development of a composite scaffold-a cross-linked and gradient scaffold that possesses unique mechanical properties that aid the functionality of tissue regeneration.

Such procedures are increasingly used in the regeneration of cartilage, heart tissue and bone. By this alone, it can be said that the original triad of tissue engineering becomes a tetrad with the introduction of mechanical signs and mechanotransduction for the most effective procedure. This area continues to be further explored with increasing external stimuli such as ultrasound and electrical impulses. It is believed that they can additionally stimulate and activate the cell to grow, multiply and establish intercellular communication for efficient functioning. A new “phenomenon” in tissue engineering is the concept of a “living scaffold”. Usually, the cell is implanted on a 3D scaffold and allows penetration into the interior. The concept of a “living scaffold” represents cells trapped inside the scaffold and permission to expand in all dimensions [89]. “Living scaffolds” that unite the concepts of nanostructured materials, growth factors, stem cells and mechanotransduction may very well be the basis for the next generation of tissue engineering.

### Regenerative Medicine and Tissue Engineering

In the last decade, regenerative medicine has developed greatly. Its main goal is to restore, maintain and improve the functions of the human body. One of the branches of regenerative medicine is tissue engineering. Through stem cells, the human body has the possibility of regeneration and recovery. Under special conditions, stem cells can create different types of cells in a certain microenvironment. Cells can be taken directly from human organs, developed from precursor and stem cells, or produced in a laboratory. Ideally, they were all taken from the patient so that the new organ would not be rejected. Tissue engineering includes the production of synthetic or natural supports, which, with their porous structure, enable the interaction, growth and development of cells, with simultaneous tissue regeneration. The carrier is then replaced by a naturally deposited extracellular matrix (ECM) during implantation in the patient's body [87].

Carriers used in tissue engineering must be biocompatible and biodegradable. The biocompatibility of the carrier implies the compatibility of the material with the biological system. After installation, the carrier must be non-toxic and must not cause an immune reaction in the body. Recently, there have been increasing demands for biodegradable materials, to avoid the permanent retention of carriers in the human body. Biodegradation of the carrier must also involve the formation of non-toxic products, as well as a degradation time that correlates with the time required for the formation of new tissue. To determine the reaction of the tissue to the foreign material and to develop the most favourable materials for making carriers, various tests, mainly in vitro methods, are carried out to obtain information about cytotoxicity, cell profiling and differentiation [90].

In vitro, tests enable the examination of carrier materials and the selection of only those materials that increase biological safety and do not have a toxic effect on cells. The basic in vitro tests are the cytotoxicity test, which tests the variability of the cells, i.e. the activity of cells on the support and staining with (3-(4,5-dimethylthiazol-2-yl)-2,5-diphenyltetrazole bromide) dye, MTT test that examines cellular metabolic activity, i.e. the viability of cells on the support [91]. The materials used to make the supports can be natural or synthetic. Natural carriers are usually made of fibroin, collagen, chitosan, casein and chitin with excellent biocompatibility, but generally very poor processability. As synthetic carriers, mostly polymer or composite carriers are used, most often carriers made of polylactide-glycolide (PLGA), polycaprolactone (PCL), polyglycolide (PGA), poly-L-lactide (PLLA), polyurethane (PU), etc. [72].

In addition, tissue engineering and nanomedicine find practical applications in the treatment of many different diseases, such as bone diseases. Some of them are caused by the loss of bone tissue due to trauma and accidents, cancer of bone tissue, bone injuries caused by firearms, amputation of limbs and osteoporosis. The excellent integration of nanoparticles in these cases gives promising results. Bisphosphonate drugs used for the treatment of osteoporosis have a high affinity for bone tissue. They can serve to connect regenerating nanoparticles and bone minerals. The synergy of bisphosphonates and regenerating nanoparticles results in a targeted therapy to promote bone tissue regeneration. Several different agents that promote osteoblast activity, as well as those that inhibit osteoclast activity, can be added to targeted therapy produced by tissue engineering. Ceramic nanoparticles are most often used for bone regeneration, i.e., poly(Lactic-co-glycolic) acid (PLGA), gelatin, collagen and chitosan.

Furthermore, nanoparticles in hydrogels have also been loaded with molecules that improve the features and effectiveness of tissue-engineered scaffolds. For example, gellan xanthan hydrogel can be used as a scaffold and the chitosan nanoparticles have been added to gellan to enhance the synthesis of growth factors responsible for bone regeneration. These growth factors, such as the bone morphogenetic protein 7 (BMP7), stimulate the stem cells to osteogenic differentiation. Heparin, the natural polysaccharide, is often incorporated in nanoparticles because its negative charge enables an electrostatic dialogue with different growth factors. Therefore, it can be used for the development of controlled systems for the transport and continuous release of growth factors, such as the complex of polymers marked as Pluronic F68. This complex has the potential to be used in cartilage regeneration after osteoarthritis [92].

Interestingly, nanomaterials such as nano clays and nanotubes have been applied in scaffolds produced by 3D printed (3DP) tissue engineering processes. Ceramic NPs have been integrated into 3DP scaffolds to improve their bioactivity. Furthermore, graphene, that is a nanostructured derived from carbon, has enabled the development of electroconductive scaffolds. For example, it was noted that PCL/reduced graphene oxide fibre scaffold improved neuronal differentiation that can be used as a perspective novel starting point for the treatment of neurodegenerative disorders. In addition, collagen is the natural biopolymer that was used in studies investigating the process of regeneration of peripheral nerves. Namely, nerve conduits containing collagen can improve the process of repairing small damages to peripheral nerves, caused by diabetic neuropathy for example [93–95]. However, these studies have mostly been conducted on animals and further research is needed to decipher the safety and effectiveness of collagen in nerve regeneration. In addition, the materials used for 3DP scaffolds still have poor biocompatibility and further research is needed for overcoming this obstacle [82, 96].

### Nanostructured Materials Forming New Tissues

Optimal conditions for tissue engineering aim to provide conditions for tissue reassembly, restoration and regeneration. Human cells can change their behaviour under controlled conditions, which has been used in tissue engineering. However, the clinical application of tissue engineering products is still limited due to many different reasons, such as the adequate source of cells, construction and mechanical properties of used scaffold and culture niche. The external conditions or microenvironment of the cell that optimize the tissue engineering process are the scaffold material, soluble factors and external physical stimulation. The scaffold material provides a 3D structure, directs and promotes cell division. The chemical, physical and mechanical properties of the scaffold material are key to optimizing the functions of a scaffold. The scaffold should have micropores and a sufficient amount of nutrients, which would allow different cells to migrate inside and proliferate, as well as the transport of waste metabolic products outside of the scaffold. This enables the survival of the integrated cells. Also, an optimal porosity of the scaffold and appropriate strength should be optimized for different tissue type that needs to be regenerated. For example, if it is used for bone regeneration, scaffold degradation should occur during a longer period and maintain the strength until the end of this process. On the other hand, if used for skin regeneration, the scaffold has to maintain its features for only about one month. Therefore, the absorption kinetics of scaffold material also has an impact on the effectiveness of tissue engineering [97].

### Support Preparation Technology

There are several technologies for preparing supports for tissue engineering and some of them are laser synthesizing, electrowashing, bioprinting, stereolithography and the like [98]. The most common process that is applied is electrowashing. The reason why this particular process is the most useful is the affordability of the price and the simplicity of the execution.

#### Electrowashing Process

Electroplating is a process in which continuous fibres from submicron to nanometer diameters are formed. This process is made possible by the use of a high-potential electric field. The advantage of this technology is its easy applicability in the laboratory and the possibility of improving and simplifying the procedure for industrial production. Fibres obtained by electrospinning have a larger specific surface area than ordinary fibres, high porosity and more intense interconnection of fibres. The greatest advantage of the electrowashing process is the controlled structure of the carrier, which enables the desired properties and functionality to be achieved for a specific application. In addition to tissue engineering, electro-washed fibres can be used as filters, wound dressings, carriers of drugs, enzymes and catalysts, protective clothing, sensors and electronic materials [99].

The basic parts of the device are a high-voltage source, a needle and a collector for collecting fibres. Due to the action of the electrostatic force created in the electric field, the fibres are formed and spun. As a result of the surface tension at the tip of the needle, a droplet of the polymer solution is formed and due to the effect of the charge induced on the surface, stretching and the formation of fibres occur. The properties and structure of the carrier depend on the parameters of the polymer solution (Concentration, viscosity, surface tension and conductivity), the process itself (Voltage, speed of fibre extraction, distance of the collector from the needle and spinning time) and environmental conditions (Temperature and humidity). Viscosity and concentration significantly affect the formation of the mat network: the lower the viscosity, the higher the tendency to create defects in the structure, while higher viscosity and higher concentration affect the formation of a more similar fibre with a larger diameter [100].

#### 3D Support Structure

The carrier must have a porous structure, with a large range between specific surface area and volume, to ensure good diffusion and migration of cells, their interaction and the permeability of nutrients and metabolic waste. Electrowashing enables the preparation of supports of 3D structure, the desired architecture, which ensures the growth of cells in a certain direction. Generally, supports with fibres with controlled orientations show better functional properties in vitro and in vivo, compared to randomly distributed fibres. Spatial geometry and size of nano- and micro-pores, as well as the distribution of fibres in the network of the carrier, influence the chemical, biological and mechanical properties of the carrier and their degradation. Additional modification of the surface of the carrier can significantly improve the antibacterial properties of the carrier, the hydrophilicity as well as the mechanical properties of the base material. Recently, there has been very intensive research on titanium dioxide, TiO_2_, considering that it shows great stability, non-toxicity, hydrophilicity, UV resistance and antibacterial properties [75].

### Bone Tissue Engineering

Bone tissue engineering differs from tissue engineering of other organs for the reason that its goal, in addition to the incorporation of new parts, is to stimulate the development of new functional tissue. From a biological perspective, to grow new bone, cells, extracellular matrix, intracellular communication, cell–matrix interaction and growth factors are needed. Bone tissue is made up of bone cells and an extracellular matrix. Bone tissue cells called osteoblasts are responsible for the production, mineralization and disposal of the bone matrix. The bone matrix consists of collagen, non-collagenous proteins, water and minerals. Hydroxyapatite is the most abundant mineral in bone tissue and more than 90% of the organic matter in bones is type I collagen [101, 102]. The process in which bone tissue is created is called osteogenesis or morphogenesis. In the formation of bone tissue, it is necessary to induce the differentiation of osteogenic cells into osteoblasts by growth factors (Principle of osteoinduction).

Osteoblasts and the created bone tissue need a suitable substrate (Principle of osteoconduction) and a porous structure that enables the three-dimensional penetration of bone tissue from the surface into the depth [101, 102]. One of the biggest challenges of orthopaedic surgery is the reconstruction of large bone damage. Only blood was transplanted more often than bone tissue. To ensure the factors necessary for bone healing and regeneration, self-transplantation or autotransplantation is very important today. The disadvantage of the procedure is an additional surgical intervention at the donor site, greater postoperative pain and potential infection. In addition to autotransplantation, allogeneic transplantation is also often used today. This is a procedure in which donor bones or bones from other mammals are used (Xenogeneic graft) [102, 103]. Such materials can cause the body to reject them because they recognize them as foreign substances in the body. An immune reaction of the organism may occur, i.e. foreign body rejection after transplantation. Artificial materials (Metal, ceramics and polymers) have a disadvantage.

**Fig. 6:**
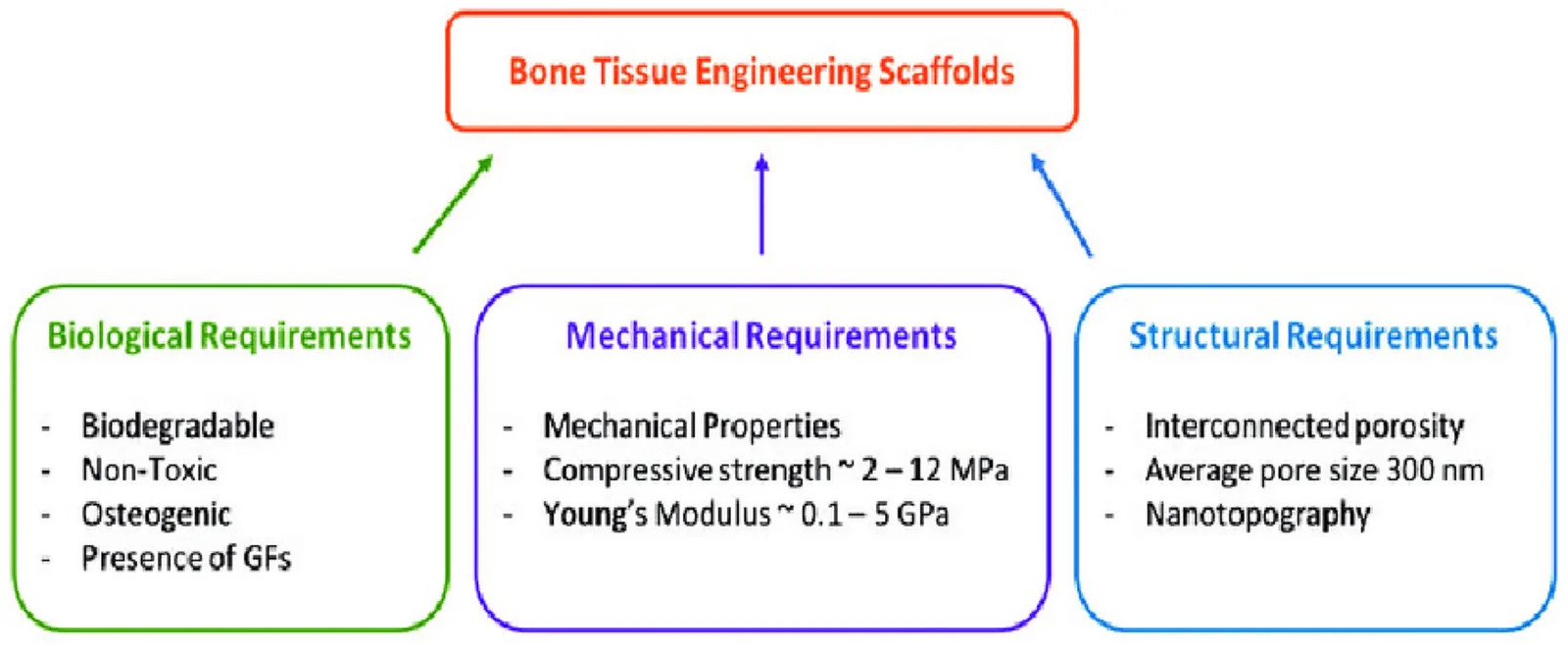
Biological, mechanical and structural properties of ideal support for bone tissue engineering [103]

The application of tissue engineering in the treatment of bone damage is a possible alternative to the classic procedures of orthopaedic reconstructive surgery. For a carrier to be ideal for use in the bone regeneration process, it must meet certain conditions. Some of the conditions are shown in Fig. 6 [104]. Special importance for the regeneration of bone tissue is given by new nanostructured porous materials obtained by a combination of bioactive ceramics and biodegradable polymers. Recently, calcium-phosphate ceramics have often been researched and the highest attention is paid to hydroxyapatite. A great advantage is given precisely to it because of its structural and great chemical matching with the inorganic component of natural bone. Characteristics such as osteoconductivity and bioactivity are some characteristics that are attractive to scientists for research in the use of tissue engineering [105].

## Future Perspectives

It can be said that currently, the most interesting area for research is nanomedicine. As noted in various examples throughout this paper, cancer is the most common disease where both detection and attempted treatment have greatly benefited from nanomedical technology. The application of nanomedicine and nanomedicine delivery systems is certainly a trend that will be researched and developed for decades to come. One major advance presented in this paper was observed in gold nanoparticles which are noticed they absorb very well soft tumour tissues making tumours sensitive to radiation-based heat [106]. Although there has been a huge interest in the advent of nanomedicine, the use of nanomedicines remains limited. Most research is conducted on animals, which requires time and resources. There are risks regarding the use of nanomedicines in humans and their impact on the environment. Looking at that, it is necessary to analyze the possible acute or chronic toxicity to both humans and the environment.

## Conclusion

Recently, nanostructured materials found to be used in targeted delivery of drug to the tissues. Another reason for the increasingly frequent use of nanoparticles and nanomaterials lies in the fact that tissues generally do not reject particles of such small dimensions. The lack of research on the process of controlled drug delivery is precise that the investigated nanoparticles are not of the same size (Some are in nanometers and some are in submicrometer). More experiments with nanoparticles and nanomaterials with one uniformity would give a better insight and a broader picture of the properties of the mentioned materials. One of the most important uses of nanostructured materials so far is in the field of tissue engineering. The main progress made in this area is the creation of implantable tissues, some of which are already in use (Skin, cartilage). The fundamental principle of tissue engineering is based on appropriate bioreactor conditions and that the 3D structure can be reassembled into a functional structure that resembles native tissue. The current goal of engineering is to create a certain specific tissue in a precise specific place through the manipulation of cells, matrices and biological factors. For such structures to be able to replace the target tissue, they must be functional and structurally and mechanically comparable to the tissue.

## Data Availability

No research data is used in the current study.
